# MRSA on surface: is it possible to control?

**DOI:** 10.1186/1753-6561-5-S6-P307

**Published:** 2011-06-29

**Authors:** CN Shimura, D De Andrade, E Watanabe, AM Ferreira, NEPECISS

**Affiliations:** 1 Escola de Enfermagem de Ribeirão Preto - USP, Ribeirão Preto, Brazil

## Introduction / objectives

The prevalence of Methicillin-resistant *Staphylococcus aureus* (MRSA) in generally is low, however may become easily resistant to multiples antibiotics. The aim of this study was to compare two disinfectants: Peractic Acid 0.1% and Etilic Alcohol 70% on surfaces contaminated with MRSA.

## Methods

This is a case control study, developed on a flowchart. Six sterile glasses surfaces (40x30cm) were contaminated with a suspension with 10^4^ cfu/ml MRSA by spreading with a sterile spatula and left dry for 10 min. The efficacy of desinfections products were measure by imprinting rodac plates with Trypticase Soy Agar holding for 1min against the surface, before and after the desinfection procedure. The glass surface was divided in three parts and each part was cleaned three times. A sterile microfibre cloth (40x38cm) made by 10% polyester, 20% polypropylene and 70% viscose were folded three times, total sixteen sides, but using nine sides only; they were moistened with 50ml of 0.1% Peractic Acid or Etilic Alcohol 70% to clean three surfaces each. Furthermore, sterile gloves were used and had their imprints (both hands) made on rodac plates, holding for 15s. Plates were incubated at 37°C for 48h.

## Results

The median of surface contamination before and after disinfection with Peractic Acid was 3.55 cfu/ml and 0 cfu/ml, for Etilic Alcohol, 4.26 cfu/ml and 0 cfu/ml respectively. Imprints of gloves after both disinfections had no growth (0 cfu/ml). The test showed a bacterial load reduction although a non-significant (p>0.05) result comparing solutions.

## Conclusion

Proceeding disinfection with Peractic Acid 0.1% or Etilic Alcohol 70% associating adequate technique had successful bacterial load reduction which contributes for environment control. Peracetic Acid has a good cost benefit however the microfibre cloth was degradating after 5 times.

## Disclosure of interest

None declared.

